# Clinical risk prediction with random forests for survival, longitudinal, and multivariate (RF-SLAM) data analysis

**DOI:** 10.1186/s12874-019-0863-0

**Published:** 2019-12-31

**Authors:** Shannon Wongvibulsin, Katherine C. Wu, Scott L. Zeger

**Affiliations:** 1 Department of Biomedical Engineering, Johns Hopkins School of Medicine, Baltimore, USA; 2 Department of Medicine, Division of Cardiology, Johns Hopkins School of Medicine, Baltimore, USA; 3 Department of Biostatistics, Johns Hopkins Bloomberg School of Public Health, Baltimore, USA

**Keywords:** Clinical risk prediction, Random forests, Survival analysis, Dynamic risk prediction

## Abstract

**Background:**

Clinical research and medical practice can be advanced through the prediction of an individual’s health state, trajectory, and responses to treatments. However, the majority of current clinical risk prediction models are based on regression approaches or machine learning algorithms that are static, rather than dynamic. To benefit from the increasing emergence of large, heterogeneous data sets, such as electronic health records (EHRs), novel tools to support improved clinical decision making through methods for individual-level risk prediction that can handle multiple variables, their interactions, and time-varying values are necessary.

**Methods:**

We introduce a novel dynamic approach to clinical risk prediction for survival, longitudinal, and multivariate (SLAM) outcomes, called random forest for SLAM data analysis (RF-SLAM). RF-SLAM is a continuous-time, random forest method for survival analysis that combines the strengths of existing statistical and machine learning methods to produce individualized Bayes estimates of piecewise-constant hazard rates. We also present a method-agnostic approach for time-varying evaluation of model performance.

**Results:**

We derive and illustrate the method by predicting sudden cardiac arrest (SCA) in the Left Ventricular Structural (LV) Predictors of Sudden Cardiac Death (SCD) Registry. We demonstrate superior performance relative to standard random forest methods for survival data. We illustrate the importance of the number of preceding heart failure hospitalizations as a time-dependent predictor in SCA risk assessment.

**Conclusions:**

RF-SLAM is a novel statistical and machine learning method that improves risk prediction by incorporating time-varying information and accommodating a large number of predictors, their interactions, and missing values. RF-SLAM is designed to easily extend to simultaneous predictions of multiple, possibly competing, events and/or repeated measurements of discrete or continuous variables over time.Trial registration: LV Structural Predictors of SCD Registry (clinicaltrials.gov, NCT01076660), retrospectively registered 25 February 2010

## Background

Clinical risk assessment has been a long-standing challenge in medicine, particularly at the individual level [1]. Questions such as “what is the probability that this patient has a particular disease?” or “what is the probability that this patient will benefit from a particular treatment?” are difficult to answer objectively but are essential in order to realize the promise of precision medicine. Accurate clinical risk prediction can help guide decision making about health status, disease trajectory, and optimal treatment plans.

Recent advances in biomedical, information, and communication technologies increase the potential to substantially improve clinical risk prediction. Modern statistical and machine learning methods are increasing our capacity to learn from a wide variety of data sources, including those that are complex, heterogeneous, and temporally-varying in nature [2–11]. Currently, most approaches to clinical risk prediction employ a small fraction of the available data. Specifically, even when variables are repeatedly measured on the same individual over time, it is common to base the patient’s risk score only on the last available measurement rather than the full history of measurements. This practice is inconsistent with the inherently dynamic nature of human health and disease; it discards valuable information from the history of the predictors, such as the rate of change of the variables or the occurrence of prior events [1].

Our work is motivated by the challenge of developing tools for clinical risk prediction that can simultaneously handle time-to-event data, repeated measurements of covariates, and repeated/multiple outcomes. We will refer to these as survival, longitudinal, and multivariate (SLAM) data. The clinical motivation for our approach is sudden cardiac arrest (SCA), a leading cause of death with complex pathophysiology that currently lacks effective tools for prediction and could benefit from methodological advances in risk assessment using SLAM data.

In this paper, we first review clinical risk prediction approaches and identify limitations of current methods. Next, we formulate the learning problem in terms of the analysis of SLAM data. Afterwards, we introduce our methodology called Random Forest for Survival, Longitudinal, and Multivariate data analysis (RF-SLAM). We then illustrate the RF-SLAM approach using the Left Ventricular (LV) Structural Predictors of Sudden Cardiac Death (SCD) Registry for SCA risk prediction and describe methodology for assessing and reporting model performance. We end with a discussion of the potential applications and extensions of RF-SLAM.

### Learning from data for clinical risk prediction

To date, most clinical risk prediction methods are based on regression approaches [1]. For example, the Cox regression model was used to develop the Framingham Risk Score [12] and logistic regression was used to develop the 30-day mortality risk prediction for patients with ST-elevation myocardial infarction [13].

Traditional regression strategies for risk prediction suffer from a number of limitations. These methods can typically only handle a small number of predictors, disregard potential interactions with time, and assume constant predictor effects throughout their entire range. As a result, the challenges not well handled by typical regression modeling strategies include: non-linearities, heterogeneity of effects (interactions), and consideration of many potential predictors. The basic assumption of a regression model is that there is a linear relationship between the risk factor and outcome. Although this can be an appropriate approximation for some risk factors, in many cases, predictors have non-linear relationships with the outcome. For example, the risk of death sharply rises with increasing age. In other cases, values both above and below the normal ranges are indicative of high risk (e.g. hypoglycaemia and hyperglycaemia, BMI for underweight and overweight individuals).

Basic regression methods also tend to assume additive relationships unless special efforts are made to identify important interactions. Nevertheless, a variable’s impact on the prediction can be influenced by another variable (e.g. gene-environment interactions, treatment-race interactions). In standard regression approaches, interactions need to be prespecified, requiring the individual developing the model to a priori include the interaction term in the model.

In addition, with a large number of potential predictor variables to consider, it is challenging to determine which to include in the model and strategies must also be taken to avoid overfitting. In the setting of missing data and many candidate predictor variables, traditional regression methods must also be paired with variable selection and missing data algorithms to accommodate large numbers of predictors and their incomplete records. When clinical risk prediction requires the consideration of a large number of predictors as well as interactions and non-linear predictor effects and missing values, moving beyond traditional regression approaches offer the potential to improve predictive performance [1]. The increasing emergence of large, heterogeneous data sets, such as electronic health records (EHRs), require novel tools for risk prediction to support improved clinical decisions. Further development of statistical machine learning approaches to address the needs of clinical risk prediction has potential to accelerate the progress towards precision medicine [1].

### Motivating Example: Sudden Cardiac Arrest (SCA) Prediction

Our work is motivated by the challenge of predicting sudden cardiac arrest (SCA), a leading cause of death with complex pathophysiology [14–17]. In the United States, each year, there are approximately 400,000 SCAs resulting in death [18]. Approximately 50% of victims do not have a prior diagnosis of cardiovascular disease and hence have limited opportunities for prevention [18]. As a result, the ability of clinicians to predict and prevent SCD remains limited.

Although the implantable cardioverter defibrillator (ICD) is considered the “cornerstone” therapy for SCD primary prevention in high risk individuals with ischemic or non-ischemic cardiomyopathy, guidelines directing their use are based upon findings of several randomized trials that have focused on dichotomizing risk based upon left ventricular ejection fraction (LVEF) [18–23]. Current guidelines define high risk as having an LVEF below 30 to 35% [18, 19]. However, LVEF is neither sensitive nor specific as an indicator for SCA. Consequently, the use of LVEF as a guide for ICD placement has resulted in poor identification of those patients most likely to benefit from implantation of an ICD and poor risk/benefit balancing of significant short and long-term complications [24]. Furthermore, applying summary results of clinical trials to individual patients can “give misleading results to physicians who care for individual, not average, patients” [25]. In fact, the rate of appropriate device firings is low (approximately 1.1 to 5.1% per year) [23, 26]. Hence, many patients are subjected to the short and long term risks of an ICD but may never require therapy and hence, receive no benefit [27].

The current limitations in methods to effectively predict and prevent SCD have been summarized in the National Heart, Lung, and Blood Institute (NHLBI) Working Group on SCD Prevention’s statement that “there is an urgent need to develop effective preventive strategies for the general population” of which effective SCA risk assessment is one important component [28, 29]. Specifically, there is a need to develop and validate SCA risk scores using phenotypic, biological, and modern biomarkers such as cardiac magnetic resonance (CMR) imaging with late gadolinium enhancement (LGE) that yields both structural and functional indices of the heart [19, 24, 30].

SCA pathophysiology is complex and requires the interaction of a vulnerable substrate and one or more dynamic triggering mechanisms to initiate and sustain a reentrant ventricular arrhythmia [30]. Accurate and precise SCA risk assessment thus requires prediction methods that handle the dynamic interplay among SCAs, triggers such as heart failure hospitalizations (HF), and other time-varying factors. Nevertheless, commonly used methods for SCA risk prediction, Cox proportional hazards model and logistic regression, do not facilitate the inclusion of nonlinear relationships or interactions among predictors when modeling with a large number of predictors [31]. Novel methods that automatically incorporate nonlinear/interaction effects and the interplay of SCA, HF, and survival (or death) could improve the accuracy and precision of SCA prediction. In particular, random forests, a decision-tree based approach, offer the advantage of relatively lower prediction error than traditional modeling approaches because of their capacity to identify complex interactions and nonlinearities of predictor effects [32–35]. However, two main disadvantages of random forests are 1) their limited interpretability and 2) to the extent of our knowledge, the inability of current random forest implementations to simultaneously handle survival, longitudinal, and multivariate (SLAM) outcomes. This research addresses these limitations through the extension of random forests for SLAM data analysis (RF-SLAM). We first begin with a description of the RF-SLAM methodology. Then, to illustrate the potential impact of RF-SLAM in clinical and translational research, we apply our approach to data from the LV Structural Predictors of SCD prospective observational registry [24, 36–41], and demonstrate the use and model performance of RF-SLAM for determining population risk as well as for predicting individualized SCA risk to guide treatment decisions.

## Methods

### Methods: random forest for survival, longitudinal, and multivariate (RF-SLAM) data analysis

To overcome the limitations of current SCA risk modeling approaches, we develop Random Forest for Survival, Longitudinal, and Multivariate (RF-SLAM) data analysis, a method that builds upon the concept of decision trees for risk stratification. Decision trees that stratify the population into strata of low and high risk based on patient characteristics are popular in medicine due to their intuitiveness and comparability to how clinicians think through clinical decisions. Nevertheless, the decision tree may “overfit” the data used to construct the tree and consequently, poorly generalize for predictions for new observations [34].

To address these issues, random forests were developed as an ensemble learning method based on a collection of decision trees, where the overall random forest prediction is the ensemble average or majority vote. Overfitting is minimized through the introduction of random selection of subjects and of predictor variables during the construction of trees in the random forest. Random sampling of predictor variables at each decision tree node decreases the correlation among the trees in the forest, and thereby improves the precision of the ensemble predictions [32]. Random forests were originally developed for regression and classification problems, but more recently, random survival forests (RSFs) have been developed for the analysis of right-censored survival data [33, 42].

#### Random survival forests (RSFs)

In this work, we expand upon randomForestSRC (random forests for survival, regression, and classification), which has previously been described [33, 42]. The key aspects of the RSF algorithm are:

1. Bootstrap the original data set to create *B* bootstrap samples.
2. On each of the *B* bootstrap samples, grow a survival tree where at each node randomly select *m*≤*p* predictors as candidate splitting variables, where *m* is the number of candidate splitting variables considered and *p* is the total number of predictors. Among the *m* variables, determine the optimal splitting variable and split point to maximize the difference between the estimated survival curves in the resulting children nodes. For RSF, the split criteria is typically based upon the log-rank statistic. Additional details are provided in the Additional file 1.
3. Continue the recursive partitioning algorithm as long as the node has no less than *d*_0_>0 unique deaths.
4. Calculate the cumulative hazard function for the terminal nodes for each tree and obtain an ensemble cumulative hazard function by averaging across the *B* trees.

Despite the benefits of RSF, limitations remain regarding the challenge of handling time-varying risk factors (e.g. heart failure exacerbations) and the interpretability of RSF predictions in the case of time-dependent outcome data. Additionally, the recent literature has expressed concerns regarding the log-rank split statistic since this is based upon the proportional hazards assumption and may suffer from significant loss of power in situations in which covariates violate the proportional hazards assumption, especially when the hazard/survival functions cross for the groups being compared [43, 44]. As a result, we introduce an extension of the random forest methodology, which we call RF-SLAM, based upon the Poisson regression log-likelihood as the split statistic to allow for the inclusion of time-varying predictors and the analysis of survival data without the restrictive proportional hazards assumption. Here, we introduce our RF-SLAM methodology for predicting survival outcomes.

### RF-SLAM methodology

**Fig. 1:**
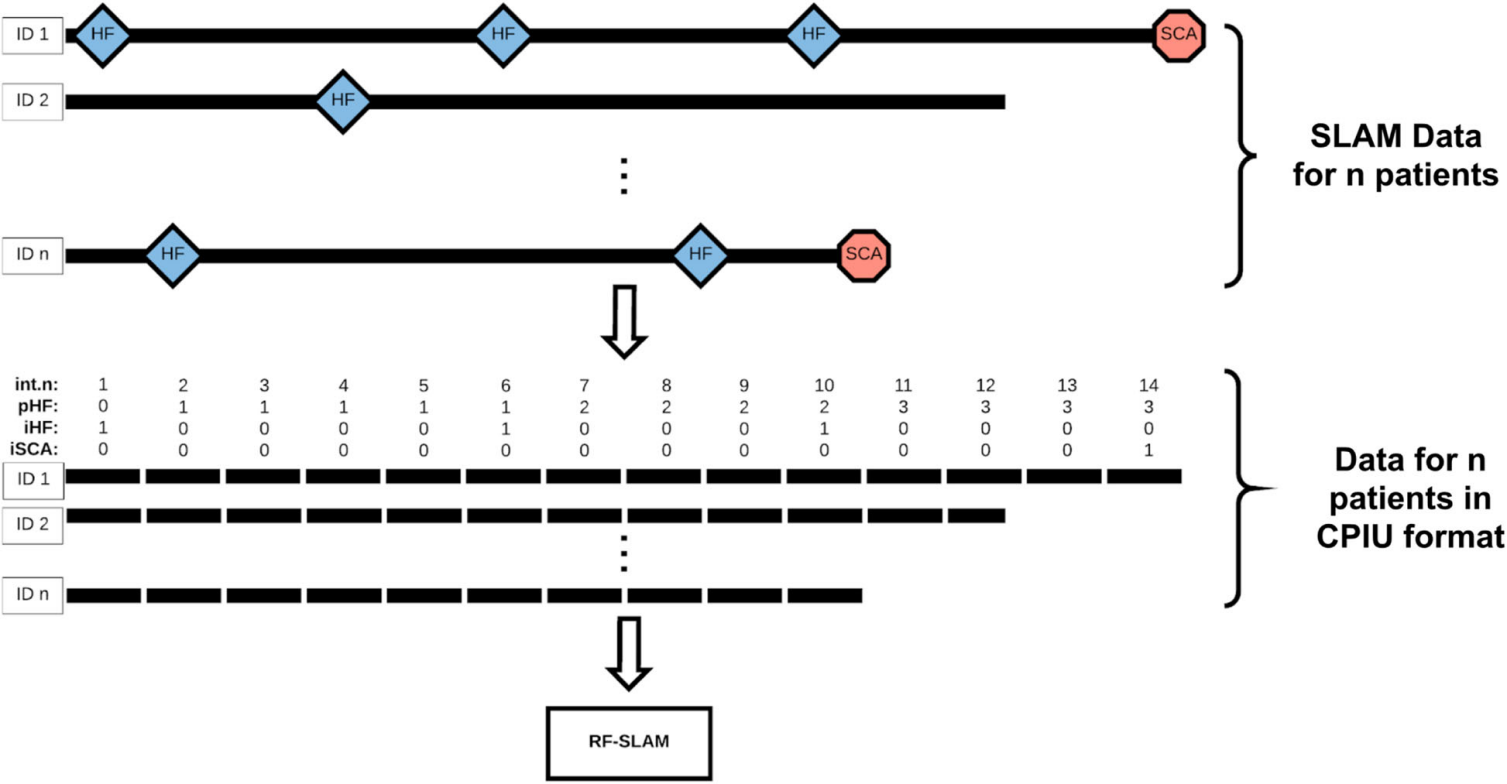
Random Forests for Survival, Longitudinal, and Multivariate (RF-SLAM) Data Analysis Overview. The Random Forests for Survival, Longitudinal, and Multivariate (RF-SLAM) data analysis approach begins with a pre-processing step to create counting process information units (CPIUs) within which we can model the possibly multivariate outcomes of interest (e.g. SCA, HF) and accommodate time-dependent covariates. For the LV Structural Predictors Registry, the time-varying covariates of interest relate to heart failure hospitalizations (HFs), indicated by the blue diamonds. In this case, CPIUs are created from the Survival, Longitudinal, and Multivariate (SLAM) data by creating a new CPIU every half year, corresponding to the frequency of follow up. The variable *int.n* represents the interval number indicating time since study enrollment in half-years. The time-varying covariates are *int.n* and *pHF* (total number of previous heart failure hospitalizations since study enrollment). Then, these CPIUs (containing the time-varying covariates along with the baseline predictors) are used as inputs in the RF-SLAM algorithm to generate the predicted probability of an SCA. The SCA event indicator is denoted with *iSCA* (0 if no event within CPIU, 1 if the event occurs within CPIU) and the heart failure hospitalization event indicator is *iHF* (0 if no event within CPIU, 1 if the event occurs within CPIU)
  CPIUs are named after the formulation of a counting process which is denoted by *N*_*e*_=*N*_*e*_(*t*), *t*≥0, where the value of *N*_*e*_ at time *t* indicates the number of events that have occurred in the interval of time (0,*t*], where *N*_*e*_(0)=0. *N*_*e*_ is nondecreasing and increases in a stepwise manner as events accumulate (i.e. *N*_*e*_ as a function of time can be modeled as a step function) [46].
  We introduce the CPIU data format and the corresponding terminology to broaden the generalizability of our approach to time-to-event analysis. The CPIU formulation of the data allows for the handling of time-varying covariates as well as multivariate count data (i.e. the counting process could be the occurrence of a single event, repeated events, or events of different types).
  Additionally, rather than considering the times-to-event as discrete times, we allow the event times to be continuous, occurring within the discrete CPIU intervals. For instance, CPIUs can be created with half-year intervals. However, the observed event times can be represented more precisely as the time at which the event occurs within the CPIU. Thus, each CPIU can potentially be of differing lengths and the length of the CPIU is recorded as the risk time. In the reformulation of the data set into the CPIU format, each subject’s data is represented as a separate record for each period of observation. Rather than having only one record per subject, the CPIU data format could result in multiple records per subject depending on the subject’s survival time and the length(s) of the observation periods defined.
  To predict the probability of an event of interest during each CPIU, we model the event probability as a Poisson process, which is a counting process that can be used to count the occurrences of the event of interest [46]. For the Poisson process, we denote *Y*(*r*) as the number of events that occurs within a time period of length *r*. Thus, *Y*(*r*)∼*Poisson*(*μ*), where *μ*=*λr* where *λ* is the event rate per unit time and *r* is the length of the time interval. We use this distributional assumption for the event probability within each CPIU as the basis for our RF-SLAM approach, which is an ensemble method based on Poisson regression trees.
- **Control of Bootstrapping:**
  Because we create CPIUs, each individual can have multiple observation intervals rather than only one as in a traditional random forest analysis. Rather than bootstrapping CPIUs, we bootstrap individuals to preserve the integrity of the original data structure. Then, we assemble together the predictions for each of the CPIUs for an individual to obtain the piecewise-constant hazard function for each bootstrap replication. This function, or the corresponding piecewise-exponential survival function, will be the basis for visualization of the risk trajectory and post-hoc analyses of how changes in different predictor variables impact an individual’s risk. By bootstrapping people rather than CPIUs and controlling the specification of the bootstrap samples on which to construct the random forest, this method also allows for the fair comparison between different random forest approaches. For example, our RF-SLAM method can be compared with RSF trained on comparable bootstrap samples, where the bootstrap samples for RF-SLAM would consist of CPIUs and the bootstrap samples for RSF would correspond to the same individuals who contributed CPIUs to the bootstrap sample.
- **RF-SLAM Splitting Criteria:**
  The RF-SLAM splitting criteria is based upon the Poisson log-likelihood below. Note, it is not necessary to directly assume that the CPIUs are independent. Rather, the process of conditioning on the past events results in a likelihood function for the discrete time hazard model under non-informative censoring that coincides with the likelihood obtained when treating the event indicators as binomial or Poisson [45].
  We use the following notation: *i*=1,2,…,*N* indicates the individual, *j*=1,2,…,*J*_*i*_ indicates the interval for individual *i*, where *J*_*i*_ is the maximum interval number for individual *i*, *μ*_*ij*_=*λ*_*ij*_*r*_*ij*_ is the expected number of events where *λ*_*ij*_ is the event rate for individual *i* during time period *j*, *r*_*ij*_ is the risk time or length of the *j*^*th*^ interval for individual *i*, *y*_*ij*_ is the number of observed events for the *j*^*th*^ interval for individual *i*, *N*_*j*_ is the total number of individuals in the risk set at the *j*^*th*^ interval, and *N* is the total number of individuals in the study.
  The RF-SLAM Poisson log-likelihood split statistic is:
  1 $$ {\begin{aligned} \sum_{i \in L} \sum_{j=1}^{J_{i}} \left[-\hat{\mu}_{ij}^{L} + y_{ij}*log\left(\hat{\mu}_{ij}^{L}\right)\right] &+ \sum_{i \in R} \sum_{j=1}^{J_{i}} \left[-\hat{\mu}_{ij}^{R} + y_{ij}*log\left(\hat{\mu}_{ij}^{R}\right)\right]\\ &- \sum_{i \in P}\sum_{j=1}^{J_{i}} \left[-\hat{\mu}_{ij}^{P} + y_{ij}*log\left(\hat{\mu}_{ij}^{P}\right)\right], \end{aligned}}  $$
  where $\hat {\mu }_{ij}^{S}$ is the estimate for expected number of events for *j*^*th*^ interval for individual *i* for node *S*, where *S*=*L*,*R*,*P* indicating the left, right, and parent nodes, respectively. The estimate $\hat {\mu }_{ij}^{S}$ is:
  2 $$\begin{array}{@{}rcl@{}} \hat{\mu}_{ij}^{S} = \hat{\lambda}_{ij}^{S}r_{ij}, \end{array} $$
  where $\hat {\lambda }_{ij}^{S}$ is the estimate of the event rate for individual *i* during interval *j*.
  Since all CPIUs within a certain node share the same event rate estimate at a given time, the Bayes estimate of the event rate for each node is defined as follows:
  3 $$\begin{array}{@{}rcl@{}} \hat{\lambda}_{j}^{S} = \frac{\alpha + \sum_{i \in S}y_{ij}}{\beta + \sum_{i \in S}r_{ij}}, \end{array} $$
  where *α*=1/(*k*^2^), where *k* can be specified by the user (we set *k*=2 as the default so the standard deviation is greater than the mean in order to capture the prior uncertainty in the estimate for $\hat {\lambda }$) and $\beta = \alpha / \hat {\lambda }$, where $\hat {\lambda }$ is the overall event rate (i.e. total number of events in the entire data set / total risk time in the entire data set) [47]. The rationale for the Bayes estimate of the event rate is provided below in the following section on the Ensemble Hazard Rate Estimates. Further details are provided in the Additional file 1.
- **Ensemble Hazard Rate Estimates:**
  Because classical approaches to estimate event rates have poor performance when there are few events (e.g. maximum likelihood estimators give overly optimistic rate estimates of zero) [48], we instead employ a Bayes estimate of the event rate. The Bayes estimate for the event rates are derived by assuming a Gamma prior for the event rates combined with a Poisson distribution for the likelihood function. Using Bayes rule, a Gamma posterior is obtained [49].
  For an observation of interest, the hazard rate estimate from each tree is obtained by sending the observation down the tree, following the branches to the left or right based upon the covariate values and decision rule at each encountered branch until the observation reaches a terminal node. Each terminal node is assigned an estimated hazard rate based upon the in-bag training data and the Bayes estimate of the event rates.
  The out-of-bag (OOB) ensemble estimate for the CPIU for individual *i* for time period *j* is obtained by averaging the estimates across the OOB trees as follows:
  4 $$\begin{array}{@{}rcl@{}} \hat{\lambda}_{e}^{OOB}(j|x_{i}) = \frac{\sum_{b=1}^{B}I_{i,b}\hat{\lambda}_{b}(j|x_{i})}{\sum_{b=1}^{B}I_{i,b}}, \end{array} $$
  where *I*_*i*,*b*_=1 if *i* is OOB for tree $\mathcal {T}_{b}$ and 0 otherwise.
  For a new observation not used in training, the estimate is based upon averaging across all the trees in the forest:
  5 $$\begin{array}{@{}rcl@{}} \hat{\lambda}_{e}(j|x_{i}) = \frac{\sum_{b=1}^{B}\hat{\lambda}_{b}(j|x_{i})}{B}. \end{array} $$
- **Missing Data:**
  Because most real data sets contain missing values, various methods for handling missing data with tree-based methods have been developed including surrogate splitting and imputing data using the proximity weighted average of nonmissing data [50–52]. Although surrogate splits can be a solution for trees, it is computationally intensive and may be infeasible when considering an ensemble of trees. With the proximity approach to data imputation, the forest is unable to predict on test data with missing values. Due to these limitations, for RF-SLAM, we adopt an adaptive tree imputation method to handle missing data based upon the approach previously introduced for RSF [33]. Overall, the idea is to impute the missing data during the tree growing process by randomly drawing from the nonmissing in-bag data within the current node. The key steps are as follows:
  1. Impute missing data prior to splitting node *h* based upon randomly drawing from the nonmissing in-bag data within node *h*.
  2. Split node *h* into two children nodes based upon the split rule.
  3. Reset the imputed data values to missing in the resulting children nodes.
  4. Repeat from Step 1 until the tree reaches the stopping criterion.
- **Data Imbalance and Terminal Node Size:**
  An additional challenge that is typical to survival data is data imbalance, where there are extreme differences between the number of censored and noncensored individuals in the study. In our situation of creating discrete CPIUs from the original survival data, the data imbalance can be seen as the predominance of *y*_*ij*_=0, corresponding to CPIUs with no events. As was proposed in Breiman’s original random forest algorithm for classification random forests, it is common to grow the trees to purity of the terminal nodes [32]. In our situation, the trees should not be grown to purity since the goal is to obtain an estimate of the hazard, or conditional probability. If the trees are grown to purity, each tree probability estimate would be 0 or 1. Instead, for hazard estimates, we retain heterogeneity in the terminal nodes by setting the default terminal node size as 10% of the total sample size based upon prior research [53, 54].

For RF-SLAM, a large number of trees (e.g. 1000) are grown to create the random forest. However, unlike with the RSF approach where the individual is the unit of analysis, RF-SLAM builds trees using data binned according to user-specified lengths of time in a format we call counting process information units (CPIUs). Each individual can have many CPIUs during the period of follow-up. For example, in the motivating SCA risk prediction problem using the LV Structural Predictors of SCD Registry, we consider follow-up time of 8 years and specify the time intervals to be 6 months long so that each individual has a CPIU representing each half-year of observation. We assume that a person’s event hazard is constant within each CPIU. This strategy allows predictor variables to change from one interval to the next. Given the partition of the follow-up time into CPIUs, we use a Poisson regression splitting criterion that does not impose the proportional hazards assumption that the predictors have a common effect across the entire follow-up time. The key aspects involved in the random forest construction using RF-SLAM are detailed below and additional information is provided in the Additional file 1:

- **Counting Process Information Units (CPIUs):** The RF-SLAM approach includes a pre-processing step where the follow-up information for each individual is partitioned into discrete segments that we refer to as counting process information units (CPIUs), as shown in Fig. 1. Specifically, each CPIU contains the following data for a prespecified bin of time: person indicator, interval indicator, multivariate outcome values (e.g. SCA and HF; 0 denoting that the event did not occur, 1 denoting that the event did occur), summary function values of outcome history, predictor values, and the length of the interval. We partition the data for each of the *N* subjects into CPIUs, which is similar to the concept of the “person-period data set” [45], to account for time-varying covariates and outcomes.

### Evaluating performance

In the era of promoting individualized health, there is growing interest in clinical prediction models that provide absolute risk estimates for individual patients. When accurate predictions are made available, they can inform clinical decisions by guiding timely action for high risk individuals who may benefit from specific preventive strategies or aggressive interventions and sparing low risk individuals from the burden of unnecessary or inappropriate interventions. Before such models are used by clinicians and patients, rigorous evaluation of their validity is essential but is often not quantified [55–59].

To characterize a model’s performance, we consider both its discrimination and calibration. For models of SLAM data, special considerations are necessary for assessing model performance. In comparison to models that are constructed to provide a prediction in a static manner (i.e. only provide a prediction for a particular time point), models fit to SLAM data are designed to be used in a dynamic manner. For instance, prognostic models are often employed for evaluating clinical risk at multiple points in time as patients return for follow-up visits and are reassessed. Naturally, the model’s performance over time may change and thus, time-varying measures of performance are necessary to assess its potential ability to serve as a clinical decision making tool [60].

#### Time-Varying AUC

The time-varying AUC is based upon time-dependent definitions of the sensitivity and specificity, as described previously [60]. These definitions take into account the dynamic risk sets. At each evaluation time there are differing CPIUs at risk for the event. The time-dependent AUC is defined as the area under the time-specific ROC curve, *ROC*_*t*_, across all thresholds *p* given by:

6 $$\begin{array}{@{}rcl@{}} AUC(t) = \int ROC_{t} (p)dp, \end{array} $$

which is equivalent to:

7 $$\begin{array}{@{}rcl@{}} AUC(t) = P(M_{l} > M_{k}|T_{l} = t, T_{k}>t), \end{array} $$

the probability that a random CPIU that experiences an event at time *t* (i.e. case) has a larger predicted value than a random CPIU that is event free (i.e. control) and also at risk at that time *t*.

We extend the approach for time-varying discrimination previously described to obtain a smooth AUC curve representing the model performance across the duration of time under consideration. Our approach consists of the following steps:

1. Calculate the $\hat {AUC}(t)$ at each time interval where $\sum \limits _{i \in N_{t}}y_{ij} > 0$, where *N*_*t*_ denotes the risk set at time *t*.
2. Calculate the estimate of the maximum variance of the $\hat {AUC}(t)$ at each of the times considered in Step 1 using the following equation:
  8 $$\begin{array}{@{}rcl@{}} \sigma_{max}^{2}(t) = \frac{\hat{AUC}(t)(1-\hat{AUC}(t))}{min\{m(t),n(t)\}}, \end{array} $$
  where *m*(*t*) is the number of cases at time *t* and *n*(*t*) is the number of controls at time *t* [61].
3. Fit a smooth curve to model the relationship between $\hat {AUC}(t)$ and time, weighted by the inverse of the variance:
  9 $$\begin{array}{@{}rcl@{}} w(t) = 1/{\sigma_{max}^{2}(t)}. \end{array} $$

Our approach for confidence intervals for $\hat {AUC}(t)$ is based upon the non-parametric bootstrap and bootstrap principle [62], which allows us to approximate how much the distribution of our AUC estimate varies around the true AUC using the distribution of how the bootstrapped AUC values vary around our estimated AUC.

#### Clinically-Relevant visualizations of discriminative ability through plots of the survival or hazard functions

In addition to plots of $\hat {AUC}(t)$ versus time, for models of SLAM data that give predictions of the hazard or survival functions, the discriminative ability of the model can also be visualized by plotting the predicted hazard or survival function versus time and color-coding the trajectory of the hazards by the observed outcomes (e.g. color-code individuals who experience the event during the study in red and all other individuals in green). The greater the separation between the predicted hazard or survival functions for individuals who experience versus do not experience the outcome, the greater the model’s discriminative ability.

#### Calibration of predicted hazard rates

Calibrating predicted hazard rates for CPIUs from SLAM data requires special consideration to account for the potential differences in risk time for each CPIU. We use two approaches to check for calibration. The first is based upon creating discrete risk groups and assessing the calibration by group. The second is based upon the Spiegelhalter’s z-statistic and does not require discretizing the data into bins.

In the first approach, to assess calibration by risk groups, groups are defined by deciles of the predicted values. For each decile, the mean predicted value is compared with the observed value. When the predicted values are hazard rates, it is important to consider the observed risk time when assessing calibration. To determine the observed hazard rate, the total number of events observed in the decile is divided by the total observed risk time for that decile. Afterwards, calibration plots with the observed versus the predicted rates can be created. In the second approach, for a formal assessment of calibration without discretizing the data into bins, we use Spiegelhalter’s z-statistic [63, 64], described further in the Additional file 1.

Confidence intervals for calibration can be formed by the non-parametric bootstrap and bootstrap principle [62]. To include confidence intervals for the calibration plots, we present the calibration results as the difference between the observed and predicted risks. Because both the predicted and observed risk for each decile can differ across bootstrap replications, rather than plotting the predicted versus observed risk, we take the difference between the predicted and observed risk and plot this difference against the corresponding decile. For well calibrated models, the confidence intervals for these differences should overlap 0, suggesting agreement between the predicted and observed values.

#### Clinically-Relevant visualizations of discrimination and calibration through plots of the survival or hazard functions

To visualize the model performance in terms of both discrimination and calibration in a clinically-relevant manner, we introduce an approach to compare the model predictions to the actually observed time-to-event data. First, we stratify patients into tertiles of predicted risk. For each group (i.e. high, intermediate, and low risk), we plot a Kaplan-Meier curve based on the observed data and compare the Kaplan-Meier curve to the predicted survival curves for individuals in the group under consideration.

### Illustrating example: sudden cardiac arrest (SCA) prediction with SLAM data

The Left Ventricular (LV) Structural Predictors of Sudden Cardiac Death (SCD) Registry is a prospective observational registry (clinicaltrials.gov, NCT01076660) that enrolled patients between November 2003 and April 2015 at three sites: Johns Hopkins Medical Institutions (Baltimore, MD), Christiana Care Health System (Newark, DE), and the University of Maryland (Baltimore, MD). Patients meeting the clinical criteria for primary prevention ICD insertion (LVEF ≤35*%*) were approached for enrollment and underwent cardiac magnetic resonance imaging (CMR) before device placement. This registry allows for the analysis of SCA risk in a clinical population with elevated SCA risk but in whom it is known that many patients will not require or benefit from ICD therapy. The design and methods of this study have been previously published, as have interim results of multivariable risk models using traditional regression approaches [24, 36–41]. The goal of the study was to identify risk factors that predispose patients to arrhythmic death. 382 patients were enrolled. The primary SCA endpoint was the occurrence of an adjudicated appropriate ICD firing for ventricular tachycardia or ventricular fibrillation or sudden arrhythmic cardiac arrest not aborted by the device. In the 8-years of follow-up, 75 individuals had the primary SCA outcome. A summary of the data is available in Additional file 1: Table S1 as well as in the published literature [24, 36–41].

Briefly, the baseline variables include information regarding demographics and clinical characteristics, risk factors, medication usage, electrophysiologic variables, laboratory values and biomarkers, LVEF by echocardiography, and CMR structural and functional indices. The time-varying covariates are the number of previous adjudicated heart failure hospitalizations and number of half-year intervals that have passed since study enrollment.

To assess the performance of our random forest method (RF-SLAM), we compare our method to the random survival forest (RSF) method currently available in the randomForestSRC (random forests for survival, regression, and classification) R package [42]. Briefly, in contrast to the RSF method, RF-SLAM can handle time-varying covariates and directly provide piecewise-constant hazard estimates (i.e. the probability of an event in a certain period of time). In our analysis of the LV Structural Predictors of SCD Registry, we compare three methods (RF-SLAM using both baseline and time-varying covariates, RF-SLAM using only baseline covariates, and RSF) for predicting SCA. Additional file 1: Table S2 provides a summary of the key differences between the three different approaches.

#### RF-SLAM

As diagrammed in Fig. 1, the first step to constructing the two RF-SLAM models is data pre-processing to create counting processing information units (CPIUs). CPIUs of half-year intervals are created since patients in this registry are followed up every six months. Thus, the maximum interval length (i.e. risk time) for the CPIUs is 0.5 years. However, if censoring or SCA occurs prior to the end of the half-year interval, the risk time is the amount of time from the start of the CPIU interval to the time of censoring or SCA. With the CPIU data format, two different random forests are constructed using RF-SLAM: one with both baseline and time-varying covariates, and a second with only baseline covariates. The parameters for the number of trees, node size, and number of variables to try at each potential split are set to be the same for both random forests. We use the typical default values of 1000 as the number of trees, 10% of the number of CPIUs as the minimum terminal node size, and the number of variables to try as the square root of the number of predictors in the model.

#### Random survival forest (RSF)

For the RSF method, we also use the default settings for the number of trees (1000), node size (15), and number of variables to try at each potential split (square root of the number of predictors in the model). After building a random forest with the RSF approach, the survival and cumulative hazard estimates can be obtained. Although the RSF method does not provide piecewise-constant hazard predictions, we develop an approach to obtain the discrete-time hazard estimates to facilitate comparisons between the methods. Specifically, the survival predictions are obtained from the RSF method and a smooth curve is fit to the predictions to obtain an estimate of the survival function. Afterwards, the value of the derivative of the log of the estimated survival function is obtained every half-year (i.e. 0.5, 1, 1.5 years, etc.) to obtain comparable hazard estimates to the RF-SLAM approach.

For all three methods, we assess the performance as described in the Methods section on model evaluation. To ensure the comparability of the bootstrap data set across the three methods, we use the same *L* boostrapped data set for all three methods and within each of the *L* boostrapped data set, we control bootstrapping by specifying a user controlled bootstrap array to ensure that the same data are used for comparable trees in the three different random forests. For the analysis presented below, we use *L*=500.

## Results

With data from the LV Structural Predictors of SCD Registry, we demonstrate a proof of concept of the RF-SLAM approach.

**Fig. 2:**
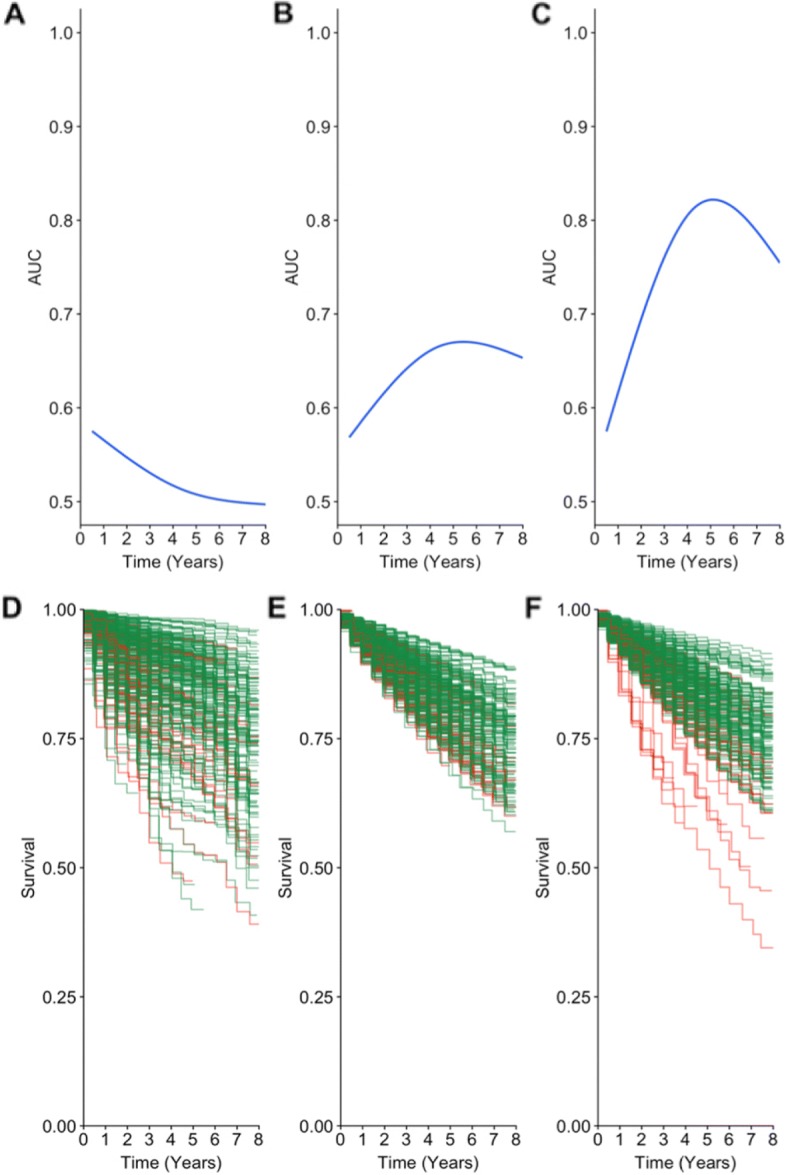
Comparison of Discrimination for Sudden Cardiac Arrest (SCA) Prediction with Different Random Forests Approaches. **a**, **b**, **c** Time-varying AUC curves for the RSF approach which uses only baseline covariates (panel **a**), RF-SLAM approach with only baseline covariates (panel **b**), RF-SLAM approach with both baseline and time-varying covariates (panel **c**). **d**, **e**, **f** Predicted survival curves from RSF (panel **d**), RF-SLAM approach with only baseline covariates (panel **e**), and RF-SLAM approach with both baseline and time-varying covariates (panel **f**). Individuals who experienced an SCA are colored-coded in red and all others are colored-coded in green. Note each column of plots corresponds to the same model (i.e. the left column corresponds to the RSF approach, center column corresponds to the RF-SLAM approach with only baseline covariate, and the right column corresponds to the RF-SLAM approach with both baseline and time-varying covariates)

Figure 2 (panels A, B, and C) shows the $\hat {AUC(t)}$ for the three different approaches. Figure 2a displays the worst performance corresponding to RSF, the random survival forest model with the log-rank split statistic and baseline covariates only. Figure 2b shows an improvement in performance with the RF-SLAM approach using the Poisson split statistic and baseline covariates only. With the inclusion of time-varying predictors in the model and the use of the Poisson split statistic, there is further improvement in model performance as measured by the $\hat {AUC(t)}$. The plots of the pairwise $\hat {AUC(t)}$ comparison between the different models along with the confidence intervals generated from the non-parametric bootstrap approach with 500 bootstrap samples are provided in Additional file 1: Figure S1.

To visualize the difference between the predictions from the different approaches, Fig. 2 (panels c, d, and e) shows the predicted survival curves, color-coded by the actual outcome. As shown in the figure, the RF-SLAM approach with both baseline and time-varying covariates (Fig. 2e) provides the best visual separation between individuals who experienced an SCA (color-coded in red) and those who did not (color-coded in green) when compared with the predicted survival curves from RF-SLAM with baseline covariates only (Fig. 2e) and RSF (Fig. 2d).

**Fig. 3:**
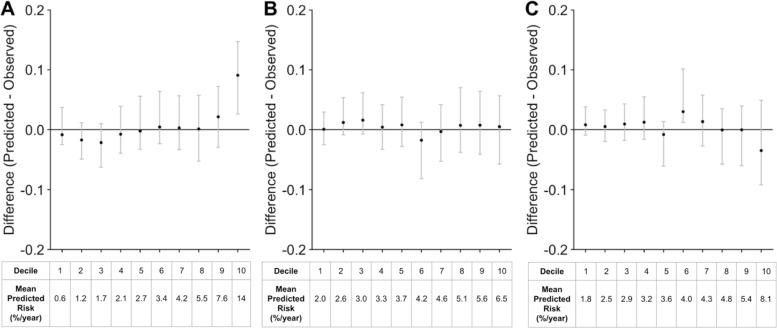
Comparison of Calibration for Sudden Cardiac Arrest (SCA) Prediction with Different Random Forests Approaches. **a** Calibration curves by decile of predicted risk for the RSF approach which uses only baseline covariates, **b** RF-SLAM approach with only baseline covariates, **c** RF-SLAM approach with both baseline and time-varying covariates. For each panel, the difference between the predicted and observed rates are plotted for each decile. The black points indicate the estimates from the original data set. The mean predicted risk (%/year) for each decile are presented at the bottom of the plot. The gray bars indicate the 95% confidence intervals from 500 bootstrapped data sets

To further assess performance, we determine the calibration. The calibration plots with the confidence intervals generated from the non-parametric bootstrap approach with 500 bootstrap samples for the three models are shown in Fig. 3. To assess calibration without discretizing into deciles, the density plot for Spiegelhalter’s z-statistic across 500 non-parametric bootstrap samples is shown in Additional file 1: Figure S2. Overall, the calibration plots suggest that the three models are well calibrated.

To visualize both the discrimination and calibration of the predictions from RF-SLAM (with both baseline and time-varying covariates), we categorize patients into different groups based upon the tertile of the average predicted risk for the individual and plot the predicted survival curves in comparison to the group’s Kaplan-Meier curve based on the actual time-to-event data observed, as shown in Additional file 1: Figure S3. These plots indicate close agreement between the predictions from RF-SLAM (with both baseline and time-varying covariates) and the observed time-to-event data as well as separation between individuals with and without the event.

## Discussion

There is growing emphasis on individualizing care based on patient-specific characteristics. The availability of large amounts of patient data and advances in computer-driven data science afford unique opportunities to implement machine learning algorithms to inform clinical decision making based on individual time-varying health trajectories and patient-specific risk profiles. With the upsurge in machine learning applications to medicine, it is imperative that such models are validated rigorously to justify clinical use.

Although the analysis of survival data for most real world applications utilize the Cox proportional hazard model, there are numerous limitations to this approach, including the restrictive assumption of proportional hazards, the need to estimate the baseline hazard to obtain hazard predictions, and the difficultly in handling a large number of variables, non-linearities, and missing predictor values. While more recently developed approaches, such as random survival forests, offer ways to overcome some of these challenges, limitations remain in areas such as handling data where predictor variables are measured longitudinally as time-varying covariates, addressing data imbalance issues, and expressing uncertainty about the predictions. To address these limitations, we introduce RF-SLAM as a piecewise-constant hazard survival analysis approach to extend the utility of random survival forests. Specifically, we develop a splitting function based upon the Poisson regression log-likelihood and Bayes estimate of the hazard rate. A comparison between RSF and RF-SLAM is provided in Additional file 1: Table S3. RF-SLAM allows for the handling of time-varying predictors and time-varying effects through the CPIU formulation of the data and the split rule based upon the Poisson loglikelihood. Additionally, since the split rule is based upon Poisson regression rather than the log-rank statistic, the splitting does not depend upon the proportional hazards assumption, which is often inappropriate or an oversimplification in the analysis of real life data. Because RSF employs the log-rank statistic for its split rule, it is possible that RSF will be unable to select potentially beneficial splits if the proportional hazards assumption is violated since the key requirement for the log-rank test optimality is proportional hazards [43, 44, 65–68].

To characterize model performance, we consider both discrimination and calibration since both are important aspects of model performance to consider in developing and evaluating a model for clinical risk prediction. When the model is intended for clinical applications, a useful model not only discriminates between individuals with and without the outcome of interest, but also provides a risk estimate that can be interpreted as a probability or a predicted rate of event occurrence (e.g. a probability of disease of 0.9 should correspond to 9 individuals having the disease out of 10 individuals who are similar to the patient under consideration, and a predicted rate of 1 event/5 years should correspond to an observation of 1 event occurring in 5 years). A highly discriminating model can be poorly calibrated and limit the clinical utility of the model when the objective is to obtain an accurate prediction of the individual’s absolute risk. Thus, appropriate model assessment is essential for the clinical impact of these prediction tools [55, 60, 69].

For demonstrating the development of RF-SLAM and its application to SCA prediction, we introduce the RFSLAM methodology with a single time-to-event outcome (i.e. time to SCA) and both static and dynamic predictors (i.e. baseline and time-varying covariates). Although not presented here, the extensions of this fundamental RF-SLAM formulation are manifold and include the consideration of multiple recurrent or competing events (e.g. repeat occurrences of SCA or the occurrences of SCA, HF, and/or death). In this work, we focus on modeling the conditional distribution of SCA given baseline information (i.e. patient demographics) and longitudinal covariates (i.e. number of previous HF hospitalizations). To handle multiple time-to-event outcomes, RF-SLAM can be extended to consider the joint distribution of these multiple outcomes through a modification of the splitting criteria of the tree construction. Furthermore, our CPIU data formulation, while used here to allow for piecewise-constant hazard survival analysis (in which the hazard is considered to be constant within each CPIU), can be easily applied to other types of analyses. For instance, for longitudinal data analysis where the outcome is a continuous variable (e.g. modeling a patient’s blood pressure trajectory over time) can be performed using the mean-squared error split function. Additionally, RF-SLAM could be used for longitudinal classification and analysis of multivariate outcomes (e.g. competing risks). Since RF-SLAM is an extension of the publicly available R package for survival, regression, and classification (randomForestSRC) [42], split functions for regression and classification are also available for RF-SLAM.

In our analysis of the LV Structural Predictors of SCD Registry, we demonstrate the best performance for SCA prediction with RF-SLAM using both baseline and timevarying covariates. Using our RF-SLAM approach with just baseline covariates also demonstrates improved predictive performance compared with RSF. While this is a univariate prediction problem and further research is required to understand the general properties of RF-SLAM, we hypothesize that the improvement in performance is due in part to the fact that our Poisson log-likelihood split function is not based upon a proportional hazards assumption and can naturally handle time-varying effects. In contrast, RSF uses the log-rank statistic, which has the key requirement of proportional hazards to achieve optimality [65–68]. In our proof-of-concept example, we demonstrate that while all three methods are well calibrated overall, the best discrimination between individuals with and without SCA is achieved with RF-SLAM using both baseline and time-varying covariates.

We also express estimates of model performance with confidence intervals through the non-parametric bootstrap [62]. Since this approach does not apply naturally to the case of expression of uncertainty for individual-level predictions because the bootstrap sample on which each forest is constructed varies, we also describe in the Additional file 1 the parametric bootstrap approach. With the parametric bootstrap approach, we develop the framework for utilizing extended data and simulated outcomes to create synthetic data sets for the quantification of the degree to which the RF-SLAM predictions might vary by training the forest on a new training set. Although other approaches, such as the jackknife and infinitesimal jackknife approaches have been applied to generate confidence intervals for random forests estimates [70], our method has utility beyond confidence interval generation. Our parametric bootstrap approach which is based upon simulating alternate training data sets can also be used in simulation studies to examine the impact of different properties of the data set on the overall predictions (e.g. the number of events, strength of the predictors, degree of data imbalance in the outcomes of interest, etc.).

Although RF-SLAM provides a new approach to the analysis of SLAM data, further research is necessary to fully understand its strengths and limitations. The data set analyzed in the proof-of-concept example is representative of the sample size and corresponding challenges often encountered in the analysis of clinical data. The width of the confidence intervals for $\hat {AUC}(t)$ (Additional file 1 Figure S1) reflect the low number of events and overall observations in the small cohort considered (75 SCA events and 382 individuals). While machine learning methods are often expected to require “big data” since they can perform well on high dimensional prediction problems with large sample sizes and large number of predictors [1], here we demonstrate that it is also possible to apply RF-SLAM to a smaller sized data set. To better understand the characteristics of RF-SLAM, its performance in data sets of varying number of patients, events, and predictors requires further analysis. Other potential areas of future work include: examining other terminal node estimates for the hazard to compare with the Bayes estimate of the hazard; comparing the performance with different node sizes, number of trees, and potential variables to consider for splitting at each node; studying the robustness of the RF-SLAM predictions to missing data; and implementing sampling methods to create balanced training data sets and determining how different implementations impact predictive performance. Other areas of work include comparing the performance of RF-SLAM with joint modeling approaches for longitudinal and time-to-event data [71–74]. Additionally, through the inclusion of error terms, joint models can account for measurement error in the covariates [71]. Future extensions of RF-SLAM that account for measurement error may be required when working with noisy data where there is high concern regarding data quality (e.g. patient generated data from self-tracking through smartphones or wearable devices).

## Conclusion

We introduce a new approach to clinical risk prediction with SLAM data that builds upon prior methods for survival analysis and tree-based strategies. RF-SLAM is a Poisson regression forest that utilizes a Poisson split rule and a Bayes estimate of the hazard rates. Our approach is distinct from the previously developed RSF for survival analysis in that RF-SLAM can handle time-varying predictors, provide a predicted probability of failure in each time interval in consideration, and quantify the uncertainty in the predictions. We also present a method-agnostic approach for time-varying evaluation of model performance. We illustrate the methods using three different proof-of-concept approaches utilizing random forests for SCA prediction in the LV Structural Predictors of SCD Registry and demonstrate the improvement in performance that can be achieved using the RF-SLAM approach with time-varying covariates. Overall, the applications and future directions of RF-SLAM are numerous and have potential to improve the analysis of data in medicine and beyond.

## Supplementary information

**Additional file 1** **Figure S1** Pairwise Comparisons of Time-Varying AUC Estimates. **Figure S2** Calibration Assessment with Spiegelhalter’s Z-Statistic. **Figure S3** Visualization of Calibration and Discrimination Through Comparison of Survival Curves by Tertile of Risk. **Table S1** Summary of Predictors in the Left Ventricular Structural Predictors of Sudden Cardiac Death (SCD) Prospective Observational Registry. **Table S2** Summary of the Three Methods Compared. **Table S3** Comparison Between RF-SLAM and RSF.

## Data Availability

The data set used and/or analyzed during the current study are available from the co-author (KCW) on reasonable request.

## Abbreviations

CMR: Cardiac magnetic resonance
CPIU: Counting process information units
HF: Heart failure hospitalizations
ICD: Implantable cardioverter defibrillator
LGE: Late gadolinium enhancement
LV: Left ventricular
LVEF: Left ventricular ejection fraction
OOB: Out-of-bag
randomForestSRC: Random forests for survival, regression, and classification
RF-SLAM: Random forests for survival, longitudinal, and multivariate data analysis
RSF: Random survival forest
SCA: Sudden cardiac arrest
SCD: Sudden cardiac death
SLAM: Survival, longitudinal, and multivariate
