# Supplementary material for: Clinical risk prediction with random forests for survival, longitudinal, and multivariate (RF-SLAM) data analysis
Source: BMC Med Res Methodol. 2019 Dec 31;20:1. doi: 10.1186/s12874-019-0863-0 (PMC6937754; doi:10.1186/s12874-019-0863-0)

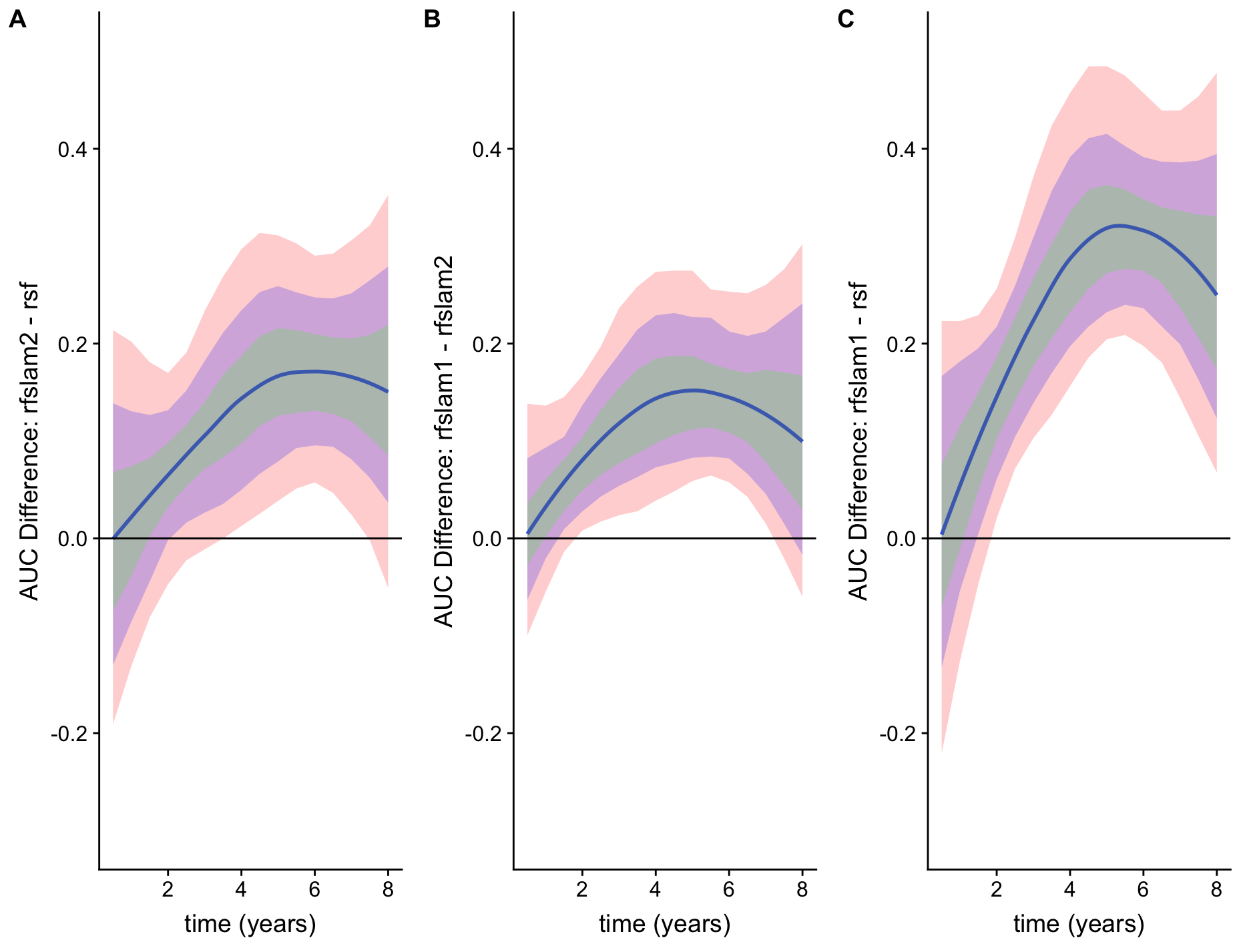


**Supplementary Figure 1: Pairwise Comparisons of Time-Varying AUC Estimates**


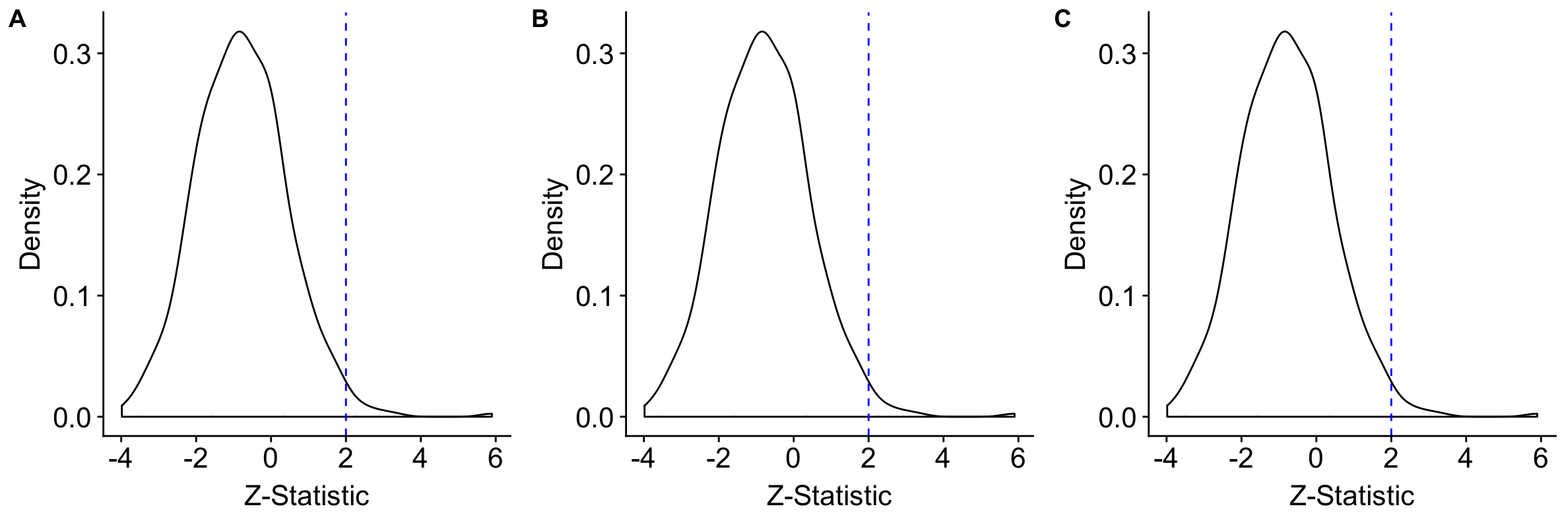


**Supplementary Figure 2: Calibration Assessment with Spiegelhalter's Z-Statistic**


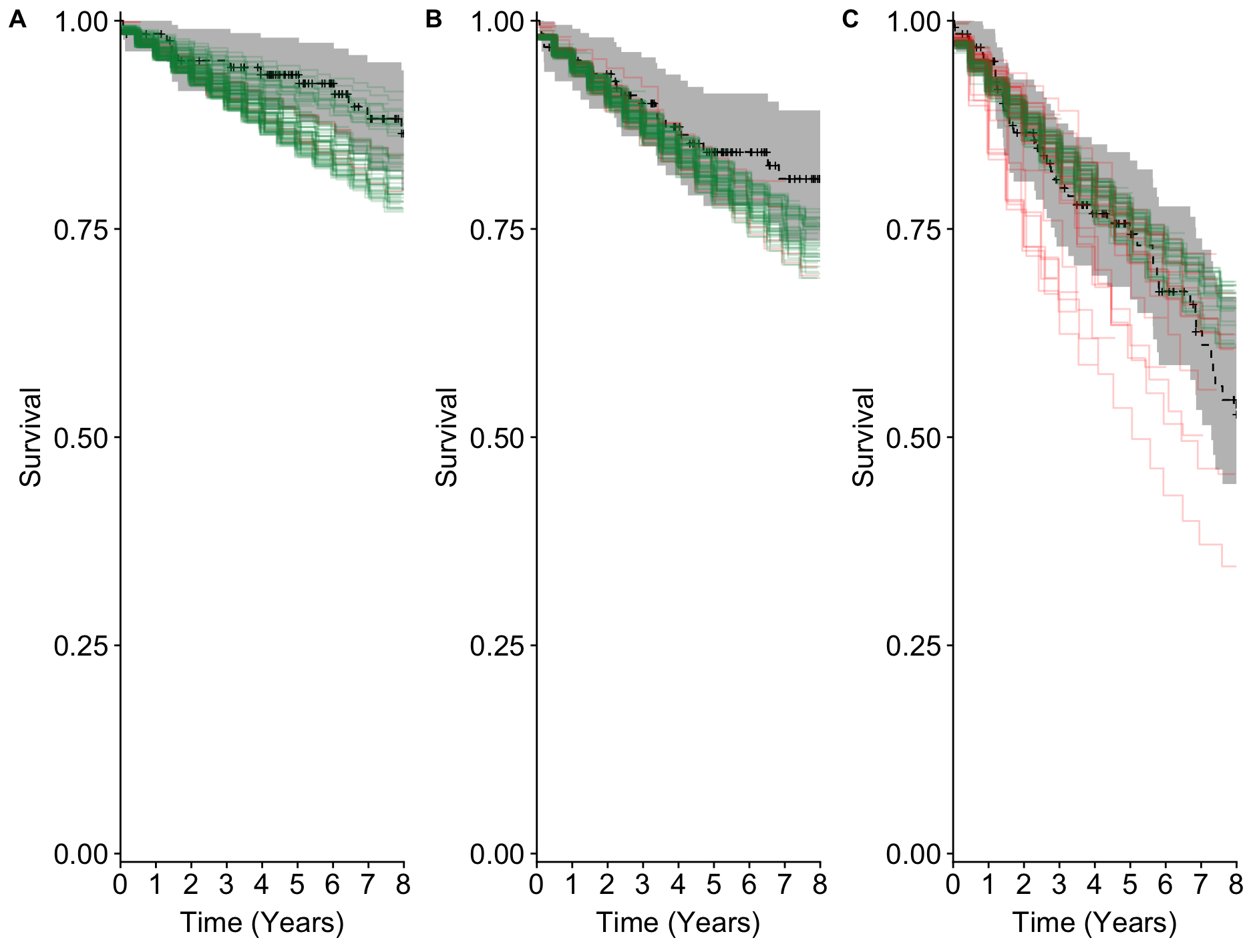


**Supplementary Figure 3: Visualization of Calibration and Discrimination Through Comparison of Survival Curves by Tertile of Risk**

**Supplementary Table 1: Summary of Predictors in the Left Ventricular Structural Predictors of Sudden Cardiac Death (SCD) Prospective Observational Registry**


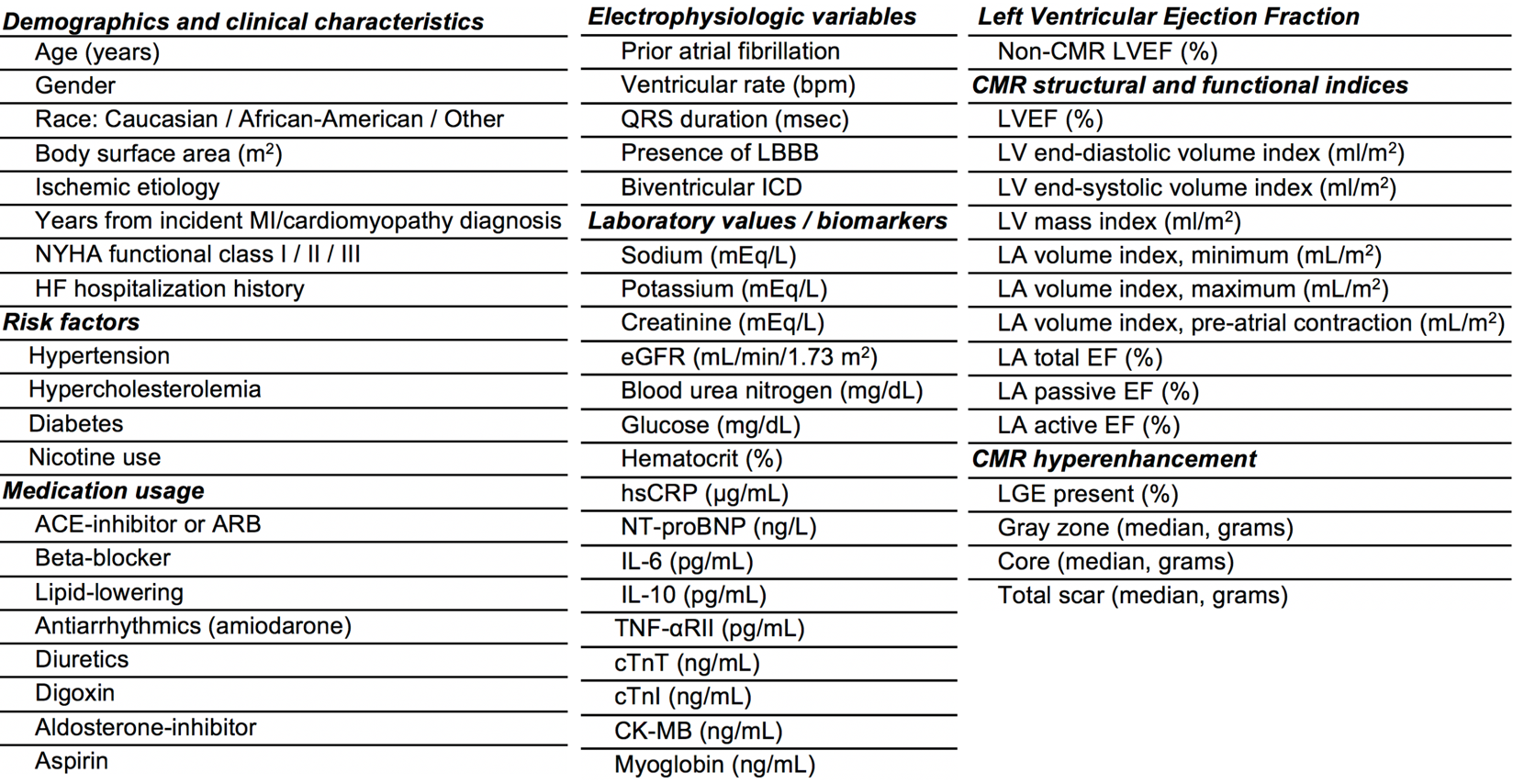


**Supplementary Table 2: Summary of the Three Methods Compared**


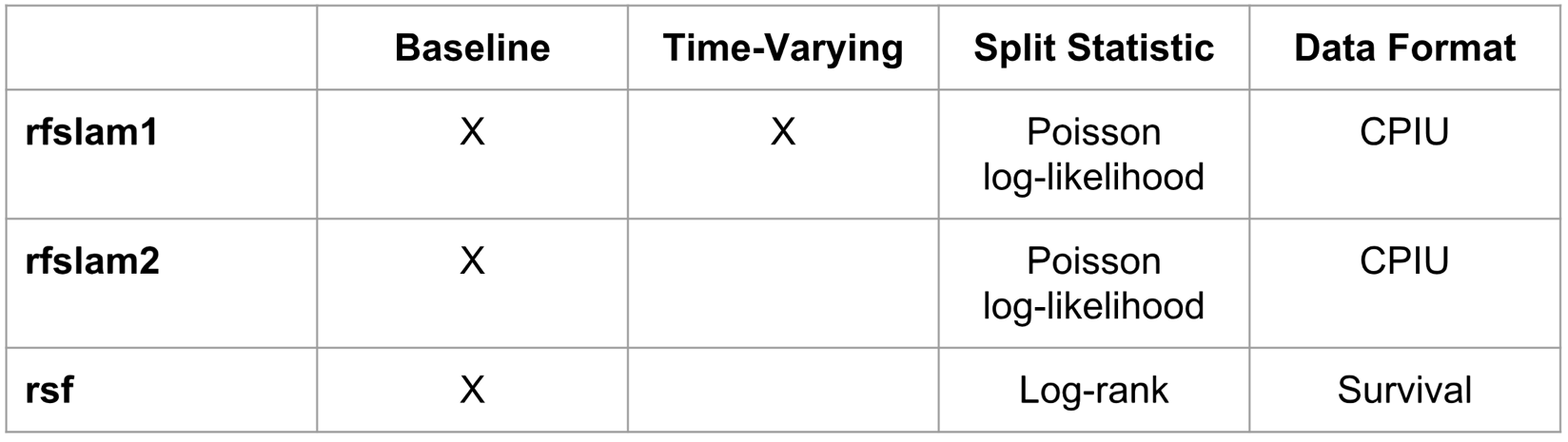


**Supplementary Table 3: Comparison Between RF-SLAM and RSF**


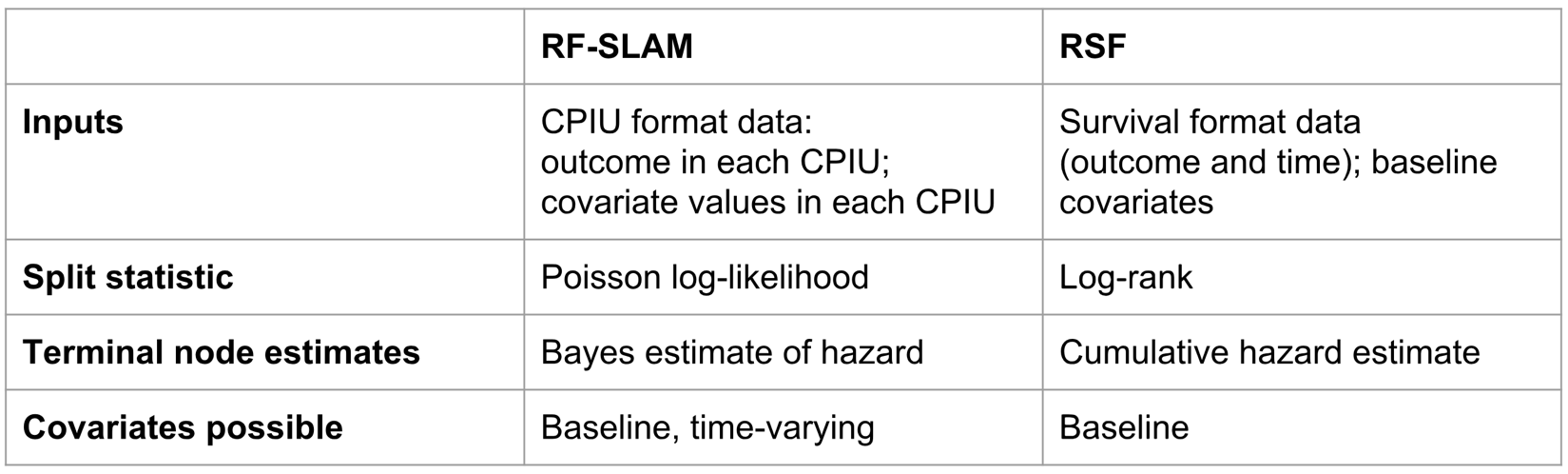

Supplement: Supplementary file 1 — Additional file 1 Figure S1 Pairwise Comparisons of Time-Varying AUC Estimates. Figure S2 Calibration Assessment with Spiegelhalter’s Z-Statistic. Figure S3 Visualization of Calibration and Discrimination Through Comparison of Survival Curves by Tertile of Risk. Table S1 Summary of Predictors in the Left Ventricular Structural Predictors of Sudden Cardiac Death (SCD) Prospective Observational Registry. Table S2 Summary of the Three Methods Compared. Table S3 Comparison Between RF-SLAM and RSF. [file 12874_2019_863_MOESM1_ESM.docx]
